# Editorial: Multimodal and Longitudinal Bioimaging Methods for Characterizing the Progressive Course of Dementia

**DOI:** 10.3389/fnagi.2019.00045

**Published:** 2019-03-14

**Authors:** Javier Ramírez, Juan M. Górriz, Stefan Teipel

**Affiliations:** ^1^Department Signal Theory, Networking and Communications, University of Granada, Granada, Spain; ^2^German Center for Neurodegenerative Diseases (DZNE), Rostock, Germany; ^3^Department of Psychosomatic Medicine, University of Rostock and DZNE Rostock/Greifswald Rostock, Germany

**Keywords:** dementia, machine learning, biomarkers, structural image analysis, functional image analysis, diagnosis and prognosis

According to the World Health Organization, in 2015 dementia affected 47 million people worldwide (or roughly 5% of the world's elderly population), a figure that is predicted to increase to 75 million in 2030 and 132 million by 2050 (World Health Organization, 2017). Dementia represents one of the major causes of disability and dependency among older people worldwide. Dementia is a broad category of mostly progressive brain diseases affecting memory, other cognitive abilities and behavior, and interfering significantly with a person's ability to maintain the activities of daily living. Alzheimer's disease (AD) is the most common cause of dementia in the elderly accounting 60–70% of cases and affects approximately 30 million individuals worldwide (Prince et al., 2013). Other major forms of dementia include vascular dementia, dementia with Lewy bodies, Parkinson's disease, frontotemporal dementia, etc.

Although new treatments are being investigated in clinical trials, no treatment to cure dementia or to alter its progressive course exists. Today, we understand that dementia appears only after a decade or more of brain degeneration (preclinical dementia) and current consensus has established the need for early recognition.

An intensive research effort is being devoted to the development of novel neuroimaging biomarkers that can provide an alert even before the cognitive decline appears. Structural and functional magnetic resonance imaging (MRI) and functional and molecular nuclear medicine neuroimaging techniques including single-photon emission computed tomography (SPECT) and positron emission tomography (PET), are widely used in combination with other blood, cerebrospinal fluid (CSF), and genetic biomarkers for early diagnosis of dementia.

Large multicenter studies are currently investigating the value of existing and novel multimodal and longitudinal neurodegeneration biomarkers. The vast amount of data available represents an opportunity for the development of more accurate statistical models of neurodegeneration enabling the early recognition as well as the characterization of the progressive course of dementia.

The aim of the Research Topic “Multimodal and Longitudinal Bioimaging Methods for Characterizing the Progressive Course of Dementia,” published in Frontiers in Aging Neuroscience, was to present the current state of the art in the theory and practice of multimodal and longitudinal neuroimaging analysis approaches for characterizing the progressive course of dementia. The Research Topic features 14 research articles. Most of the contributions analyzed disease progression and the relationships among underlying pathological changes.

Differentiating between Parkinson's disease (PD) and atypical parkinsonian syndromes (APS) is still a challenge, specially at early stages when the patients show similar symptoms. During last years, several computational approaches have been proposed in order to improve the diagnosis of PD, but their accuracy is still limited (Segovia et al., 2015, 2017). The first paper of the Research Topic is devoted to the development of analysis methods for diagnosis of idiopathic Parkinson's disease (IPD), multiple system atrophy (MSA), and progressive supranuclear palsy (PSP) (Guevara et al.). Ten healthy controls, 20 IPD, 39 PSP, and 41 MSA patients were studied using MRI and Structural Imaging Evaluation with Normalization of Atrophy (SIENA) (Smith et al., 2002).

Bipolar disorders such as the Late Onset Bipolar Disorder (LOBD) is often difficult to be differentiated from neurodegenerative dementias due to common cognitive and behavioral impairment symptoms. In a multimodal study Besga et al. determined differences in white matter (WM) tract integrity between AD and LOBD cases, and their correlation with systemic inflammatory, neurotrophic factors, and oxidative stress blood plasma biomarkers. Differences in WM tract integrity reflected greater behavioral and mood clinical features of LOBD and together with alterations of neuroinflammatory blood markers, different impact of neuroinflammation in both diseases.

The paper by Yap et al. focuses on visualization of hyperactivation in neurodegeneration based on prefrontal oxygenation showing a comparative study of mild AD dementia, mild cognitive impairment, and healthy controls. Functional near-infrared spectroscopy (fNIRS) signals were analyzed together with a semantic verbal fluency task (SVFT) to investigate any compensation exhibited by the prefrontal cortex (PFC). It was shown that the task-elicited hyperactivation in MCI might reflect the presence of compensatory mechanisms, and hypoactivation in mild AD dementia could reflect an inability to compensate.

Several works have suggested that multimodal data analysis has the potential to improve the diagnosis of dementia (Ortiz et al., 2018). The paper by Höller et al. showed that combining quantitative markers from SPECT and EEG increased discrimination of MCI and AD cases from people with depression, and that the resulting diagnostic accuracies were higher than the diagnostic accuracy of each single modality alone.

The paper by Guan et al. addresses the development of MCI subtype classification techniques to enable early intervention with targeted treatment. A sample of 184 community-dwelling individuals (aged 73–85 years) was analyzed and cortical surface based measurements were computed from longitudinal and cross-sectional MRI scans. Their results using feature selection and a voting classifier suggested that longitudinal features were not superior to the cross-sectional features for MCI subtype classifications.

The paper by Sarica et al. provides a systematic review of random forests (Ramírez et al., 2009, 2010, 2018) as an enabling machine learning technique for automatic early diagnosis and prognosis of AD using single and multi-modal neuroimaging data.

Emerging imaging modalities are also covered in this Research Topic. Simultaneous EEG-fMRI acquisitions allow combining the spatial resolution of fMRI with the temporal resolution of EEG. The paper by Brueggen et al. carried out a study of simultaneous fMRI-EEG acquisitions in a sample of AD patients and controls and showed a reduced positive association between alpha band power and BOLD fluctuations in AD patients, compared to the control subjects. ^18^F-DMFP-PET is a neuroimaging modality used to diagnose Parkinson's disease (PD) by examining postsynaptic dopamine D2/3 receptors. Segovia et al. proposed a novel methodology to preprocess ^18^F-DMFP-PET data that improves the accuracy of computer aided diagnosis systems for PD. PET data were segmented into 4 maps according to the intensity and the neighborhood of the voxels using an algorithm based on Hidden Markov Random Field. Then, the maps were individually normalized so that the shape of their histograms could be modeled by a template Gaussian distribution. The results outperformed those reported by previous approaches.

The article by Alderson et al. used a multimodal approach to assess white matter integrity between thalamus and default mode network (DMN) components and associated effective connectivity in healthy controls (HCs) relative to aMCI patients. Their methodology enabled the DMN of each subject to be identified using independent component analysis (ICA) and resting state effective connectivity that was calculated between thalamus and DMN nodes. Significant changes in the diffusivity metrics of thalamic white matter projection tracts to hippocampus, posterior cingulate cortex and lateral inferior parietal lobe were identified.

Based on the notion that amyloid may induce neuronal network hypersynchony in eary AD stages the work by Mueller and Weiner developed a graph and cluster analysis on a sample of Florbetapir-F18 PET and task-free 3T functional and structural MRI and found distinct pattern of hypersynchrony with underlying white matter connectivity in amyloid positive vs. negative cognitively normal older subjects.

Mutation carriers may exhibit distinct neuropathological features of neurodegenerative diseases. As an example, patients with frontotemporal dementia (FTD) or amyotrophic lateral sclerosis (ALS) due to a C9orf72 mutation are characterized by two distinct types of characteristic protein depositions. The study by Schönecker et al. aimed to determine if mutation carriers showed an enhanced degree of thalamic and cerebellar atrophy compared to sporadic FTD and ALS patients or healthy controls.

The paper by Salvatore et al. analyzed progression of AD using a machine learning method in a cohort of 200 subjects obtained from the Alzheimer's Disease Neuroimaging Initiative (ADNI). Subjects were followed-up for 24 months, and grouped as AD, progressive-MCI to AD, stable-MCI, and cognitively normal (CN). Structural T1-weighted MRI and neuropsychological measures were used to train a classifier to distinguish mild-AD patients (AD + progressive MCI) from subjects with a benign cognitive course (stable-MCI + CN). Principal component analysis (PCA) and partial Least squares (PLS) were used as feature extraction methods similar to previous studies (López et al., 2011; Segovia et al., 2012; Khedher et al., 2015).

^18^F-labeled amyloid tracers have been approved with similar efficacy to PIB and longer half-life: ^18^F-florbetapir in 2012, ^18^F-flutemetamol in 2013 and ^18^F-florbetaben (FBB) in 2014. Based on the broad availability of PET-CT scanners, the work by Segovia et al. proposed to include in the analysis the information about gray matter neurodegeneration provided by CT images in order to improve the diagnosis of AD. Specifically, standardized uptake values (SUVs) from 18F-FBB PET data were obtained using only voxels belonging to gray matter in CT images. The results suggested that SUVs calculated according to the proposed method allowed AD and non-AD subjects to be more accurately differentiated. This agrees with previous studies on the use of structural MRI scans to correct amyloid PET data for spill out effect of signal from gray matter to CSF and for spill in effect from white to gray matter (Gonzalez-Escamilla et al., 2017).

Previous works have shown that beta-amyloid, tau, neuroinflammation and neurodegeneration all play a significant role in the etiology of Alzheimer's disease (AD) (Lehmann et al., 2013). In Su et al. a novel computational modeling approach for multimodal MRI and PET inspired by reaction rate equation in chemical kinetics is proposed to investigate the progression of AD and relationships among underlying pathological changes. The study is motivated by the fact that the relationship between them is often unclear, mainly because the time scale associated to dementia generally exceeds the one of other studies and the challenge of observing the ordering of the pathological changes during the progression of the disease.

In summary, the Research Topic provides a transnosological, transdisciplinary view on current developments in neuroimaging techniques and their application to neurodegenerative dementias. It depicts a dynamic landscape of emerging acquisition and analyses techniques that share, however, three key features:

- The combination of modalities within and beyond imaging techniques to improve group separation and modeling of longitudinal decline
- The combination of data driven and model driven analysis approaches to get the best from both worlds: full exploitation of available data and top down restriction of the outcome space based on a priori assumptions on disease pathogenesis
- Related to these features, the ability to test assumptions of underlying disease mechanisms in combinatorial multimodal imaging analyses, crossing even the boundaries of single disease entities.

Such highly interdisciplinary approach serves as blueprint not only for future research in neurodegenerative dementias but in other neuropsychiatric diseases as well.

## Author Contributions

JR, JG, and ST equally participate in the preparation of this manuscript.

### Conflict of Interest Statement

The authors declare that the research was conducted in the absence of any commercial or financial relationships that could be construed as a potential conflict of interest.
